# Medicine in Paris

**Published:** 1840-07

**Authors:** 


### THE WESTERN JOURNAL.

No. 1.

LOUISVILLE, JULY 25, 1840.

### MEDICINE IN PARIS.

We have the pleasure of laying before our readers another letter from our esteemed correspondent, Dr. Linton, giving an account of the state of medicine in the French capital. It will be found interesting, though Dr. L. apologises for not making it so practical as he intends his future communications shall be. He proposes shortly visiting Edinburgh and Dublin, from which points he promises we shall often hear from him. Y.

Paris, May 1, 1840.

*Dear Sir:*—In my last letter, I attempted to afford you a concise account of the organization of medical institutions in Paris, and France generally. I proceed, in the present, to a notice of the actual state of the science—a task to which my limits would prevent me from doing ample justice, not to speak of other and weightier reasons. Since the downfall of Broussaisism, no other exclusive system of medicine has been favored with the general sanction of the profession. The time seems to have gone by when system-builders may expect the multitude to bow down to their idols, and yield a slavish credence to their dogmas and dreams. The doctrine of comparative equality appears to be obtaining, as well in the scientific, as in the political world, and as the notions of the divine rights of kings are about being forever banished from the latter, the yoke of the authority of great names is almost thrown off by the former.

In medicine, this is unquestionably the result of the great facilities which are now afforded to all, for the study of its various departments. Every one can examine for himself the facts and truths whose logical deductions should constitute the principles of the profession; and is of course in a condition to think and reason, as well as observe for himself; or in other words, to build theories for himself—not airy and speculative hypotheses, but theories, rational theories, which is but another term for cautious inductions, and the truths—the principles, which flow necessarily from them. *Exclusive theory* in medicine, will, I think, be found in every instance to be the offspring of hasty induction or generalizations. The pathologist, for example, finds that irritation is capable of exciting inflammation, and a host of diseases, local and general; and in the ardour of his discoveries, hastens to the conclusion, that all "the ills that flesh is heir to," are thus produced. Another observes, that, in the forms of fever called typhus, the small intestines are the seat of a peculiar species of ulcers; and this fact being observed in a greater or less number of cases, he arrives at the general conclusion, that this lesion is not only an invariable accompaniment of typhus, but the *fons et origo* of the malady.

Hahnemann observed, or is said to have observed, that quinine given to the healthy subject produces intermittent fever; and knowing, that this therapeutic agent is almost a specific in this disease, he concluded, after making other analogous observations, that the best method of curing disease is to administer, in each particular case, the agent capable of producing a *similar* disease. Now, there is *some* truth in this. We know that cold is a therapeutic agent of undoubted power even in the diseases which it produces. What is better for a frozen limb than frictions with snow? The same law, says Hahnemann, holds good in the prevention of disease, which is best effected by substituting the mild for the graver forms; and the small pox may be cited as an example. Is not the process of acclimation effected in the same way? But Hahnemann went still further. In a state of disease, says he, the organs are far more irritable than in health, and therefore the dose of the agent administered must be greatly diminished. Hence his small, almost infinitesimal doses. Examples of this hasty generalization, or formation of theories, might be multiplied were it necessary. But I only wished to express and illustrate the opinion, that all the exclusive theories which have, from time to time, been presented to the medical public, consist of inductions pushed too far—deductions more or less logical, with a goodly amount of speculation, and that consequently they are not altogether either true or false.

Thousands of diseases are produced by irritation. This no one doubts; though the theory, that all the morbid changes to which the organization is subject are thus produced, may well be questioned or rejected; and thus, though *Broussaisism* may be regarded as fallen, the facts which formed the basis of his too aspiring edifice still stand secure and unmoved.

Though no exclusive system of medicine prevails at present in France, yet it cannot be said, that the science is "in ruins." The observation of facts is going on rapidly; their classification, and the deduction of principles from them is the work of calmness and caution. One class is not permitted, like the rod of Aaron, to swallow up the rest, and thus constitute a huge, though frail, and indefensible body, known by the name of "exclusive theory." The humoral pathology is gaining ground in Paris. You hear almost as much about lesions of the blood, as lesions of the solids—as much about passive congestions and sedatives, as irritations and inflammations; and of sustaining the patient by cordials and stimulants, as of reducing him by the lancet, leeches and gum water. No *exclusive* theory of pathology or therapeutics need be expected again, in France, from the fact, that no such system can be true. The industrious and talented observer may be disposed, occasionally, to push his conclusions farther than the facts warrent; but in such an event, instead of being hailed by the multitude as an extraordinary genius, and of seeing his mental offspring adored as the veritable child of philosophy and truth, hundreds of observers as industrious and gifted as himself will not only refuse their credence to his dogmas, but respond in resistless might, thus far, and no farther, the facts justify you in going, and here shall thy proud spirit of speculation be stayed."

Thus *Chomel* and *Rostan* watch the movements of *Louis* and *Bouillaud*—thus *Gibert* and *Trousseau* expose the errors of the younger *Broussais* and *Piory*—thus the materialist combats the aërial philosophy of the vitalist; and the rationalist, the uncertainty and unsoundness of the principles of the numerist; whilst the thoughtful Andral regards the whole with unbiased scrutiny. Thus, also, in the department of surgery, the innovations of *Civiale Guerin* and *Leroy d'Etiolles*, are submitted afresh to the crucible of experiment by *Velpeau*, *Roux*, *Sanson*, and a host of others; whilst *Lisfranc*, the fault-finding and unsparing demi-god of French Surgery, pours out upon each new principle, method, and procedure, the vials of relentless criticism. Thus, in short, are the masterspirits of the times engaged in close observation and cautious induction, each ready to expose the hasty generalizations of the others. In such a state of things, he does not understand the signs of the times, who looks for the establishment of an exclusive system. He may look for what is daily taking place—the perfectionment of the many principles or generalizations of the profession; but not for the time when one simple generalization shall constitute the science of medicine.

It is almost unnecessary to say, that all parties agree as to the importance of anatomical studies: Pathological anatomy is cultivated with indefatigable zeal; though it is not to be denied, that something more is necessary in order to cure diseases, than the mere knowledge of the pathological lesions in which they consist; and that a great many of those lesions, though grave enough to produce death, elude the most scrutinizing autopsies. Alterations in the organization, which consist merely in a loss of equilibrium between the solids and fluids, and those especially which affect the nervous centres, though of a serious and sometimes promptly fatal character, are not easily detected in *post mortem* examinations. These considerations have caused many to estimate pathological anatomy beneath its deserts; whilst, on the other hand, its advantages are perhaps overrated by those devoted to its cultivation. It is clear however, that every thing is not to be expected from any one source; and that this department of medical science presents its quota of light to the physician. The *numerists*, at the head of whom may be placed

Bouillaud, regard numbers as the great lever which is to raise medicine to the height of certainty. Fifty cases of typhus, say they, were treated by the lancet, and fifty by purgatives and stimulants: out of the former, forty recovered; out of the latter, but twenty; therefore, the one treatment is superior to the other in the proportion of forty to twenty, or two to one. In short, they rely greatly on statistical tables. Now, who disputes the value of statistics in medicine? No one, I suppose; but, at the same time it is easy to perceive, that they are not infallible, when we consider the great variety presented by the constitutions of patients, and that indeed no two cases of disease are exactly alike.

I had intended, in this letter, as I promised in my last, to give something more practical. I find, however, that I have almost exhausted my paper without having complied with that promise. I will, therefore, conclude by giving you the classification adopted by *Cruveilhier* in his course of pathological anatomy: I transcribe from 'the black board.'

Lesions Morbids.—1st *Divisions.*—2d *Adhesions.*—3d *Displacements.*—4th *Canalization.*—5th *Hypertrophy.*—6th *Transformations.*—7th *Hypersecretions.*—8th *Hæmorrhagy.*—9th *Inflammation.*—10th *Tubercle.*—11th *Cancers.*

The professor commenced his present course upon the fourth order of his classification, viz: *Canalization*, under which he treats of all the alterations of canals—as of the digestive tube, the excretory ducts, the vascular system, &c., &c. He has been ever since his appointment to this chair engaged with the three foregoing orders, at which rate he will be some years in getting through his entire course. I suppose he intends it to be a *complete* one. In my next, I will endeavor to give a resumé of the lectures of Professor *Trousseau*, on the therapeutic properties of iron. He is regarded as one of the ablest therapeuticians of the capital.

